# Transcriptomic and cellular decoding of scaffolds-induced suture mesenchyme regeneration

**DOI:** 10.1038/s41368-024-00295-y

**Published:** 2024-04-23

**Authors:** Jiayi Wu, Feifei Li, Peng Yu, Changhao Yu, Chuyi Han, Yitian Wang, Fanyuan Yu, Ling Ye

**Affiliations:** 1 State Key Laboratory of Oral Diseases & National Center for Stomatology & National Clinical Research Center for Oral Diseases & West China Hospital of Stomatology, Sichuan University, Chengdu, China; 2 State Key Laboratory of Oral Diseases & National Center for Stomatology & National Clinical Research Center for Oral Diseases & Department of Cariology and Endodontics, West China Hospital of Stomatology, Sichuan University, Chengdu, China; 3 State Key Laboratory of Oral Diseases & National Center for Stomatology & National Clinical Research Center for Oral Diseases & Department of Pediatric Dentistry, West China Hospital of Stomatology, Sichuan University, Chengdu, China

**Keywords:** Regeneration, Mesenchymal stem cells

## Abstract

Precise orchestration of cell fate determination underlies the success of scaffold-based skeletal regeneration. Despite extensive studies on mineralized parenchymal tissue rebuilding, regenerating and maintaining undifferentiated mesenchyme within calvarial bone remain very challenging with limited advances yet. Current knowledge has evidenced the indispensability of rebuilding suture mesenchymal stem cell niches to avoid severe brain or even systematic damage. But to date, the absence of promising therapeutic biomaterials/scaffolds remains. The reason lies in the shortage of fundamental knowledge and methodological evidence to understand the cellular fate regulations of scaffolds. To address these issues, in this study, we systematically investigated the cellular fate determinations and transcriptomic mechanisms by distinct types of commonly used calvarial scaffolds. Our data elucidated the natural processes without scaffold transplantation and demonstrated how different scaffolds altered in vivo cellular responses. A feasible scaffold, polylactic acid electrospinning membrane (PLA), was next identified to precisely control mesenchymal ingrowth and self-renewal to rebuild non-osteogenic suture-like tissue at the defect center, meanwhile supporting proper osteointegration with defect bony edges. Especially, transcriptome analysis and cellular mechanisms underlying the well-orchestrated cell fate determination of PLA were deciphered. This study for the first time cellularly decoded the fate regulations of scaffolds in suture-bony composite defect healing, offering clinicians potential choices for regenerating such complicated injuries.

## Introduction

As fibrous connections between cranial bones, cranial sutures serve as joints enabling slight skull movements while providing a protective cushion for the brain during stressful periods.^1^ In humans, most cranial sutures, except the frontal suture, fuse between the ages of 26 and 30, with additional closure activity from the fifties to the late seventies.^2^ This suggests that complicated calvarial defects often encompass suture destruction. In such cases, the absence of sutures, which provide exclusive niches for mesenchymal stem cells (MSC)^3–9^ destroys craniofacial bone homeostasis and impairs the inherent regenerative capacity, resulting in delayed union and non-union of calvarial defects.^3,4,6,7,10^ It is reported that the speed of calvarial defect healing is inversely linked to the distance between the cranial suture and the injury site.^10^ Furthermore, the removal of both coronal and sagittal sutures results in the non-healing of parietal bone defects.^6^ Therefore, the restoration of suture mesenchyme emerges as a pivotal aspect of treating calvarial defects.

At present, cranioplasty continues to be the predominant approach targeting calvarial defects.^11–14^ Nonetheless, regardless of whether using autografts, allografts, or artificial scaffolds, the emphasis has consistently been the restoration of cranial hard tissues, neglecting the critical need for the reconstruction of suture mesenchyme. Regarding suture regeneration, investigations are conducted via the utilization of suture-containing bone flaps or MSC. After transplantation into calvarial defects, bone flaps with sutures integrate effectively, whereas those without sutures exhibit non-union and an inability to generate new periosteum, dura mater, or osteocytes.^3^ In further in vivo studies, it is observed that suture transplants maintain suture patency and facilitate ongoing ossification at the recipient site.^15–17^ Meanwhile, there is evident regeneration and even excessive growth at the donor sites, implying the prospective applicability of suture transplants.^15,16^ MSC grafting has long been established as an effective approach for calvarial defects.^4,18,19^ MSC in sutures rapidly transition into a proliferative state and migrate to the injury site in response to calvarial defects.^3,4^ Implanting suture-derived MSC into the defects significantly accelerates the bone healing process.^4^ Besides, the transplantation of MSC isolated from bone marrow into sagittal suture-bony composite defects resulted in suture-like gap formation.^20^ Notably, a recent study highlights the successful regeneration of suture-bony complex using Gli1^+^ MSC, contributing to intracranial pressure modulation, skull deformities correction, and neurocognitive function enhancement.^21^ Even so, when applied to humans, both suture transplants and MSC may encounter limitations associated with supply constraints, immune rejection, and ethical concerns.^22^

With the development of regenerative medicine and biomedical engineering, researchers are progressively drawn to tissue engineering scaffolds for their versatility and customizable properties.^23^ Noteworthy is the utilization of MSC-loaded gelatin methacryloyl hydrogel (GelMA) and polylactic acid (PLA)-co-glycolic membrane, documented for their success in osseous healing and suture regeneration.^20,21^ As for non-cell-loading scaffolds, the efficacy of polytetrafluoroethylene in achieving similar regenerative outcomes is recognized but largely unexplored beyond histological analysis, highlighting a gap in deeper mechanism studies.^24,25^ Prior studies emphasized the precise regulation of cell fate determination as the key to maintaining the physiological structure of the cranial suture-bony complex.^8,9^ For example, the imbalance between MSC proliferation and osteoblast (OB) differentiation results in pathological suture expansion or fusion.^8,26^ An absence of fundamental knowledge and methodological evidence on the scaffold’s control on cellular fate exists, hindering the development of optimal therapeutic biomaterials. Thus, our focus was directed toward the cellular responses of the scaffolds following transplantation into suture-bony composite defects. To date, numerous scaffolds of distinct types have been utilized in repairing calvarial defects including GelMA,^21,27^ porous chitosan (CTS) scaffold,^28^ and PLA electrospinning membrane.^29,30^ Despite this, a big inadequacy exists in exploring their regenerative potential on suture mesenchyme and their specific mechanisms of cellular destiny regulation.

In this study, we investigated three kinds of commonly used tissue engineering scaffolds for calvarial healing and comprehensively decoded their cellular effects on fate determination during calvarial mesenchyme regeneration. Specifically, we focused on how these biomaterials mediated the ingrowth of intrinsic cells and regulated crucial cellular processes including lineage commitment, self-renewal, stemness preservation, and signal responses.

## Results

### Study design and characterization of the candidate scaffolds

**Fig. 1:**
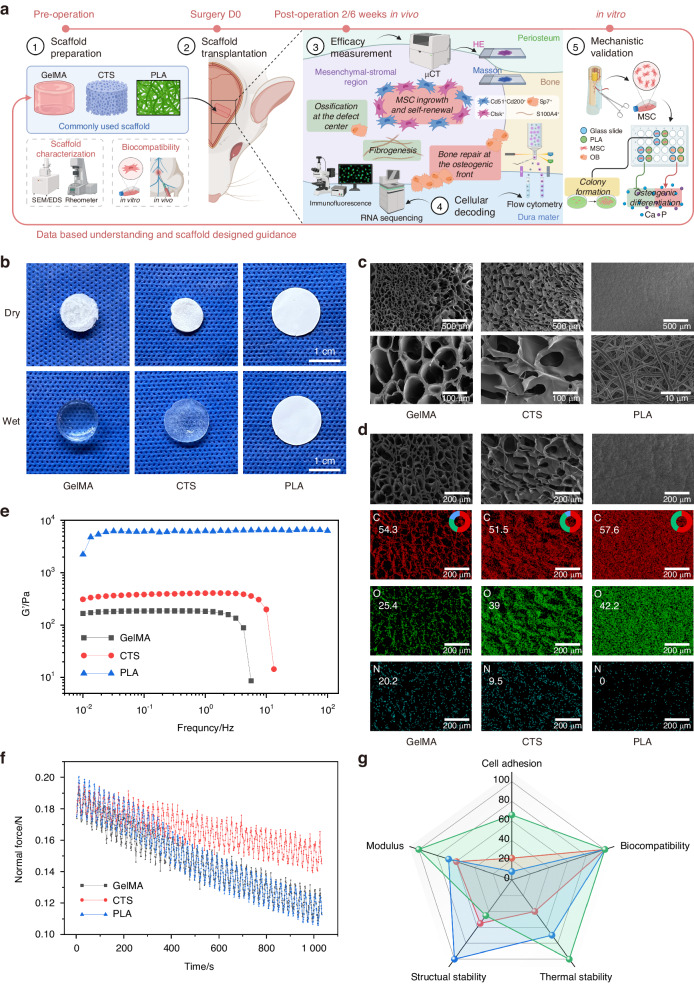
Study strategy and characterization of the candidate scaffolds. **a** The flowchart of this study (created with Biorender.com). MSC, mesenchymal stem cells; OB, osteoblasts. **b** Photographs of GelMA, CTS, and PLA in dry and wet conditions. **c** SEM displaying the microstructures of GelMA, CTS, and PLA. **d** EDS elemental mapping of carbon (C), oxygen (O), and nitrogen (N) elements in the corresponding SEM images. The pie charts and the numbers in the images display the elemental proportions. Frequency sweeps (**e**, storage modulus G’ vs frequency) and mechanical cycle testing (**f**) of GelMA, CTS, and PLA scaffolds. **g** Comprehensive performance of each scaffold. In the radar chart evaluation, multiple criteria including (relative) cell adhesion, biocompatibility, (relative) modulus, thermal stability, and (relative) mechanical stability were considered to compare the performance of each scaffold

To analyze the cellular regulations of the candidate scaffolds and provide fundamental guidance for scaffold design targeting suture-bony composite defects, a series of investigations were conducted. The detailed flowchart of this study is displayed in Fig. 1a. For the characterization of candidate scaffolds, photographs depicted the general appearance in both dry and wet conditions (Fig. 1b). Scanning electron microscopy (SEM) observations showed the porous microstructures of GelMA and CTS under lyophilized conditions (Fig. 1c). In a hydrated state, GelMA formed a compact gel, whereas CTS formed a physically porous gel (Fig. 1b). Unlike these two, PLA existed in the form of electrospinning membranes (Fig. 1b, c). Energy-dispersive X-ray spectroscopy (EDS) mapping indicated that GelMA and CTS comprised C, O, and N elements, whereas PLA contained only C and O elements (Fig. 1d). The mechanical properties were assessed through frequency sweeps and cycle testing (Fig. 1e, f). Figure 1e demonstrated that the storage modulus remained consistent as the frequency increased, suggesting the formation of stable network structures in the three scaffolds,^31^ among which PLA exhibited the highest storage modulus (Fig. 1e, g). CTS displayed the most favorable performance under cyclically applied external forces, indicating its superior structural stability compared with the other two (Fig. 1f, g). For thermal stability, thermogravimetric analysis (TGA) results showed GelMA and CTS with weight reduction peaks below 100 °C (Fig. S1a). In vitro degradation tests indicated no degradation of CTS and PLA in phosphate-buffered saline (PBS) at 37 °C during the 8 weeks (Fig. S1b). Combining the findings of these two, the thermal stability order of the three scaffolds was PLA, CTS, and GelMA (Fig. 1g). As for cytotoxicity in vitro, MSC could adhere to and proliferate on the scaffolds, with the highest cell adhesion count on PLA, followed by GelMA and CTS (Fig. 1g and S2a, d). No adverse influence of scaffolds was observed on the survival of co-cultured MSC by live-dead assays (Fig. S2b, c). In terms of biosafety in vivo, measurements of rat body weight, head length, head width, cranial length, and cranial width 6 weeks post-surgery revealed no significant differences among the groups (Fig. S3a). Meanwhile, microscopic examination of histological images exhibited the absence of evident tissue harm or pathological alterations across primary organs (Fig. S3b). Moreover, blood routine and biochemical analysis were performed, revealing no substantial alterations attributable to the scaffold implantation (Fig. S3c). Collectively, these findings confirmed the excellent biocompatibility of the three scaffolds (Fig. 1g).

### Phenotypic screening identified PLA as suture mesenchyme-regenerative scaffolds

**Fig. 2:**
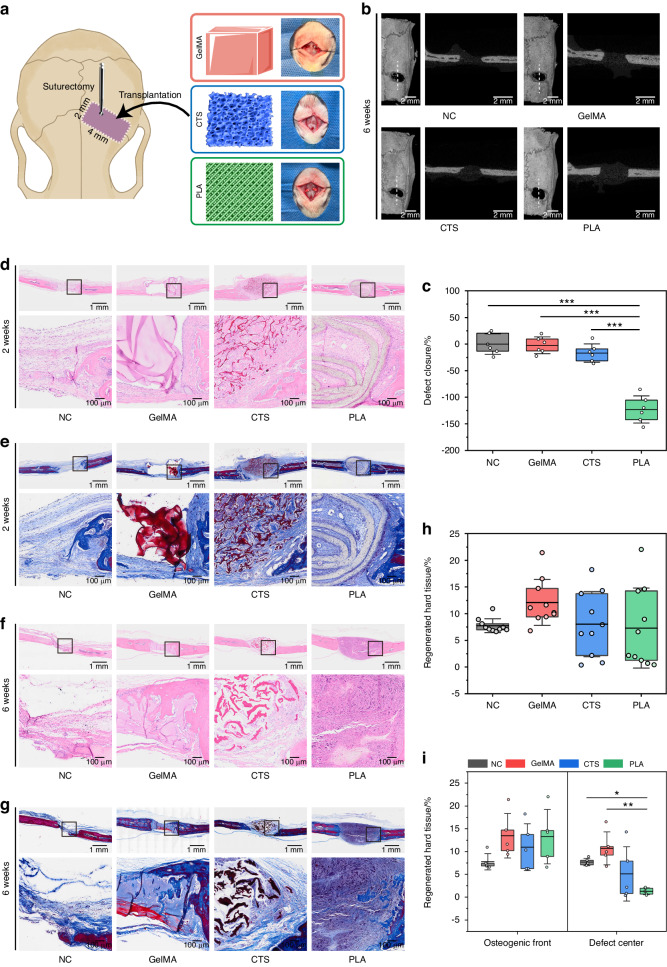
Phenotypic performance of the scaffolds at animal and histological levels. **a** Schematic illustration of a suture-bony composite defect across coronal sutures (a rectangular defect = 2 mm × 4 mm) in SD rats. **b** Representative µCT images and cross-sectional views of suture-bony composite defects 6 weeks postoperatively. The cross-sections depict the locations indicated by the white dashed lines in 3D images. **c** Quantitative analysis of defect closure 6 weeks post-surgery through µCT assessment. n = 6 replicates/group. H&E (**d**, **f**) and Masson’s trichrome staining (**e**, **g**) of suture-bony composite defects after scaffold implantation for 2 weeks (**d**, **e**) and 6 weeks (**f**, **g**). The high-magnification images are from the selected regions (black boxes) in the low-magnification images. **h**, **i** Statistical data for (**g**). Blue-stained mineralized fibers and/or mature bone were quantitively analyzed. Statistical analysis was conducted for the entire 10 randomly selected regions (**h**), followed by separate analyses for 5 osteogenic front views and 5 defect center views (**i**). NC, suture-bony composite defects without scaffold implantation. Data are expressed as mean ± SD. *P < 0.05; **P < 0.01; ***P < 0.001

To preliminarily compare the overall performance of the scaffolds in vivo, 2 × 4 mm rectangular defects were generated along the coronal sutures of rats, followed by the transplantation of scaffolds (Fig. 2a). In Sprague-Dawley (SD) rats, apart from the posterior frontal suture, all the other sutures remain open the whole life.^32^ However, concerning the current surgical model, a complete fusion of the coronal suture was observed 6 months postoperatively (Fig. S4a, b). This is consistent with the reported risk of cranial suture loss following calvarial defects,^20^ necessitating interventions to regenerate mesenchymal tissue and maintain suture patency. 6 weeks after scaffold implantation, micro-computed tomography (µCT) images and accompanying quantitative data displayed an inhibitory tendency on suture closure in PLA (Fig. 2b, c). Further histological analysis revealed persistent, undegraded GelMA and CTS components within the defects (Fig. 2f, g). Conversely, PLA displayed near-complete degradation by 6 weeks (Fig. 2f, g). Cellular infiltration was observed along the direction of PLA folds at the 2-week time point (Fig. 2d, e) accompanied by blue-stained nuclei penetrating the spinning’s interior (Fig. 2e). At 6 weeks, NC (suture-bony composite defects without scaffold implantation) and GelMA exhibited mineralized fibers and mature bone at the defect center, indicating possible suture closure risks in the future (Fig. 2g). In contrast, PLA featured abundant nascent mesenchymal tissues in the defects (Fig. 2g), suggesting capabilities to restore mesenchymal tissue and maintain suture patency. To quantify the regenerated hard tissue, 5 regions from the osteogenic front and 5 regions from the defect center of Masson’s images (Fig. 2g) were randomly selected (Fig. 6a). The statistical analysis of the total 10 regions revealed no significant differences in mineralized fibers and bone quantity among groups (Fig. 2h). However, when assessing separately, PLA exhibited a notable reduction in central ossification, while no significant distinctions were observed at the repairing forefront (Fig. 2i). Based on these animal and histological detections, PLA was preliminarily speculated to have potential in suture-mesenchyme regeneration.

### Transcriptomic analysis verified the mesenchyme-regenerative capacity of PLA

**Fig. 3:**
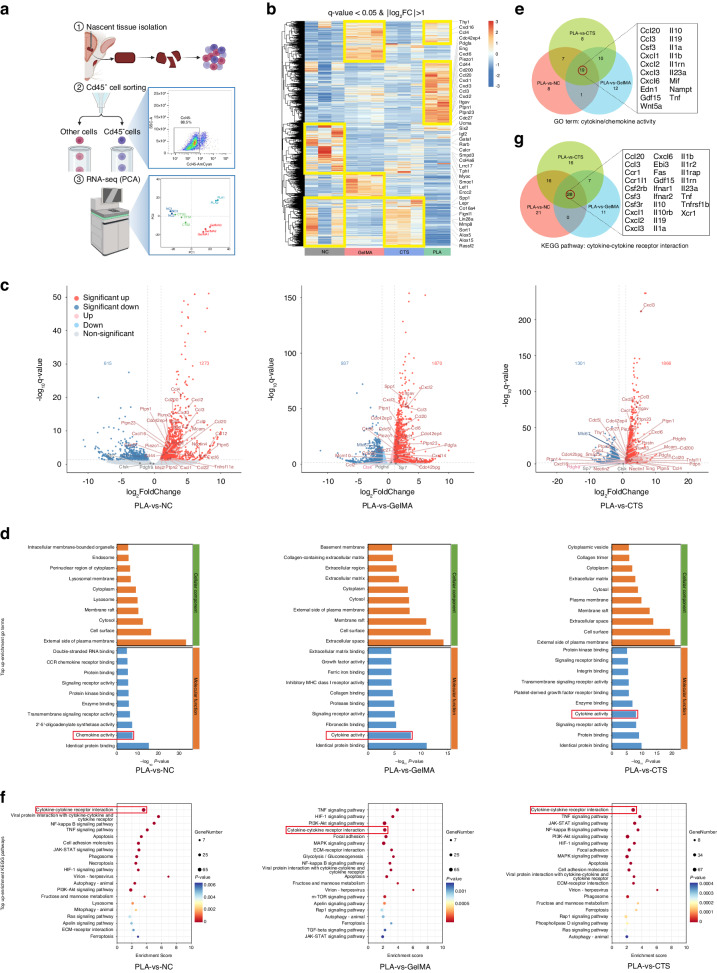
Transcriptome analysis of scaffold-mediated MSC recruitment. **a** Workflow of the RNA-Seq experiment with three major steps: nascent tissue isolation, Cd45^-^ cell sorting (Gating strategy of FCM for Cd45^-^ cells), and RNA-seq analysis (PCA, Principal component analysis). **b** Heatmap analysis of DEGs. **c** Volcano plot illustrating the DEGs in PLA compared with NC, GelMA, and CTS, respectively. The upregulated DEGs and other genes of interest are labeled. **d** The top 20 up-enrichment GO terms of cellular component and molecular function in the PLA group compared with NC, GelMA, and CTS groups. **e** Venn diagram displaying common upregulated DEGs in chemokine/cytokine activity (GO:0008009 and GO:0005125) of the three comparisons (PLA-vs-NC, PLA-vs-GelMA, and PLA-vs-CTS). **f** The top 15 up-enrichment KEGG pathways in the PLA group compared with NC, GelMA, and CTS groups. **g** Venn diagram illustrating common upregulated DEGs in cytokine-cytokine receptor interaction (rno04060) of the three comparisons (PLA-vs-NC, PLA-vs-GelMA, and PLA-vs-CTS). NC, suture-bony composite defects without scaffold implantation

To well know the efficacy of PLA in regenerating mesenchyme and inhibiting ossification at the transcriptome level, we subjected Cd45^-^ mesenchymal lineage cells, sorted from digested nascent tissue (Fig. 3a), to RNA sequencing (RNA-seq). The findings revealed significant differences among sample groups, while within-group variations were minimal (Fig. 3a and S5a–c), indicating distinct biological functions of each scaffold. The heatmap illustrated the distinctive expression pattern of differentially expressed genes (DEGs) among the groups (Fig. 3b). Of note, PLA demonstrated a significantly higher number of upregulated DEGs, with counts of 1273, 1870, and 1866 compared to NC, GelMA, and CTS, respectively (Figs.  3c and S5d). Among these upregulated DEGs, we observed a spectrum of stemness-related markers such as *Cd200*, *Itgav* (*Cd51*), *Cd44*, *Eng*, and *Thy1* (Fig. 3c),^8,33^ providing indications of PLA’s capacity for MSC recruitment. Furthermore, the FPKM (fragments per kilobase of transcript per Million mapped reads) values of genes associated with chemokines and cytokines exhibited an increase in PLA (Fig. 3c). Concurrently, chemokine/cytokine activity and cytokine-cytokine receptor interaction emerged as top up-enrichment GO (Gene Ontology) terms and KEGG (Kyoto Encyclopedia of Genes and Genomes) pathway for PLA (Fig. 3d, f). 19 upregulated DEGs in chemokine/cytokine activity (Fig. 3e) and 28 upregulated DEGs in cytokine-cytokine receptor interaction (Fig. 3g) were identified in all three comparisons (PLA-vs-NC, PLA-vs-GelMA, and PLA-vs-CTS), highlighting the pivotal role of PLA in establishing a microenvironment for cell communication mediated by chemokines and cytokines, thereby contributing to the active mesenchyme regeneration.

**Fig. 4:**
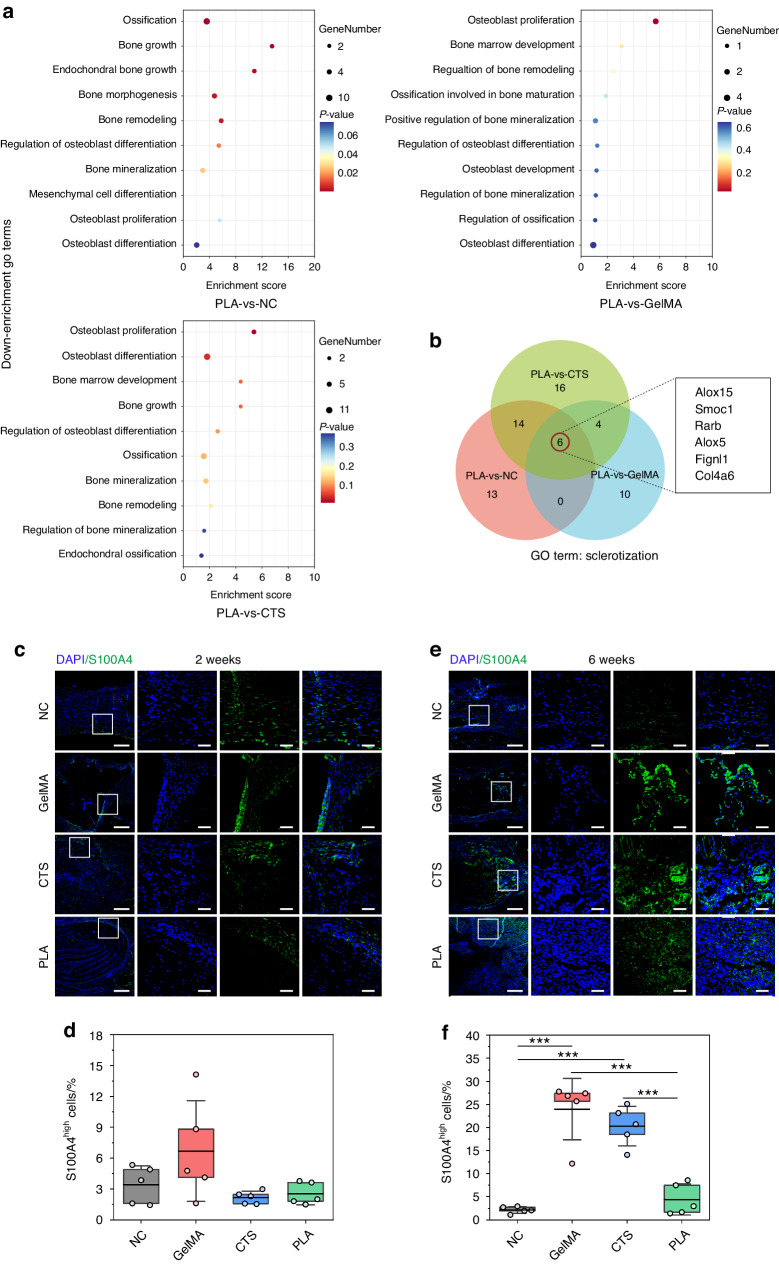
Transcriptome analysis of ossification and cellular decoding on fibrogenesis. **a** The down-enrichment GO terms related to ossification in the PLA group compared with NC, GelMA, and CTS groups. **b** Venn diagram of common downregulated DEGs associated with sclerotization in the three comparisons (PLA-vs-NC, PLA-vs-GelMA, and PLA-vs-CTS). IF images of fibroblast marker S100A4 (in green) in defect regions at 2 weeks (**c**) and 6 weeks (**e**) post-surgery. The high-magnification images are from the selected regions (white boxes) in the low-magnification images. Scar bar, 250 μm in low magnification and 50 μm in high magnification. **d**, **f** Statistical data for (**c**, **e**), respectively. NC, suture-bony composite defect without scaffold implantation. Data are expressed as mean ± SD. **P < 0.01; ***P < 0.001

As for tissue ossification, GO enrichment was applied to assess the function of downregulated DEGs related to sclerotization (Fig. S5e). Comparative analysis with NC revealed a downregulation in biological processes such as ossification, bone growth, endochondral bone growth, and bone morphogenesis in PLA (Fig. 4a). The consistent downregulation of ossification by PLA, in comparison with other scaffolds, demonstrated a decrease in OB proliferation compared to GelMA and a reduction in OB proliferation and differentiation compared to CTS (Fig. 4a). Additionally, 6 downregulated DEGs associated with sclerotization were uncovered in all three comparisons (PLA-vs-NC, PLA-vs-GelMA, and PLA-vs-CTS). All these align with the observed phenotype of inhibited diffuse ossification in the center of suture-bony composite defects implanted with PLA.

### Cellular decoding indicated no inductive fibrogenesis by PLA in suture-bony composite defects

Fibrosis, defined as the excessive accumulation of extracellular matrix components, signifies an adverse outcome of various organ injuries such as the heart, lung, skin, liver, kidney, and bone.^34,35^ Scaffold implantation may trigger fibrogenesis at the interface, impeding tissue integration and causing permanent scar restoration.^36^ To eliminate this problem, we employed immunofluorescence (IF) staining with S100A4, a distinctive fibroblast marker commonly employed to monitor tissue fibrosis,^37^ on tissue sections. The findings revealed that the restorative process of NC did not inherently generate substantial fibrous tissue (Fig. 4c–f). At 2 weeks post-surgery, only limited fibrous tissue was observed within the defects of each group (Fig. 4c, d). However, by 6 weeks, improper scaffolds like GelMA and CTS significantly triggered fibrogenesis (Fig. 4e, f). Unlike these two, PLA did not induce the formation of fibrotic mesenchymal tissue, with no significant difference in the proportion of S100A4^high^ cells compared to NC (Fig. 4e, f).

### Cellular decoding displayed enhanced MSC ingrowth and self-renewal by PLA

**Fig. 5:**
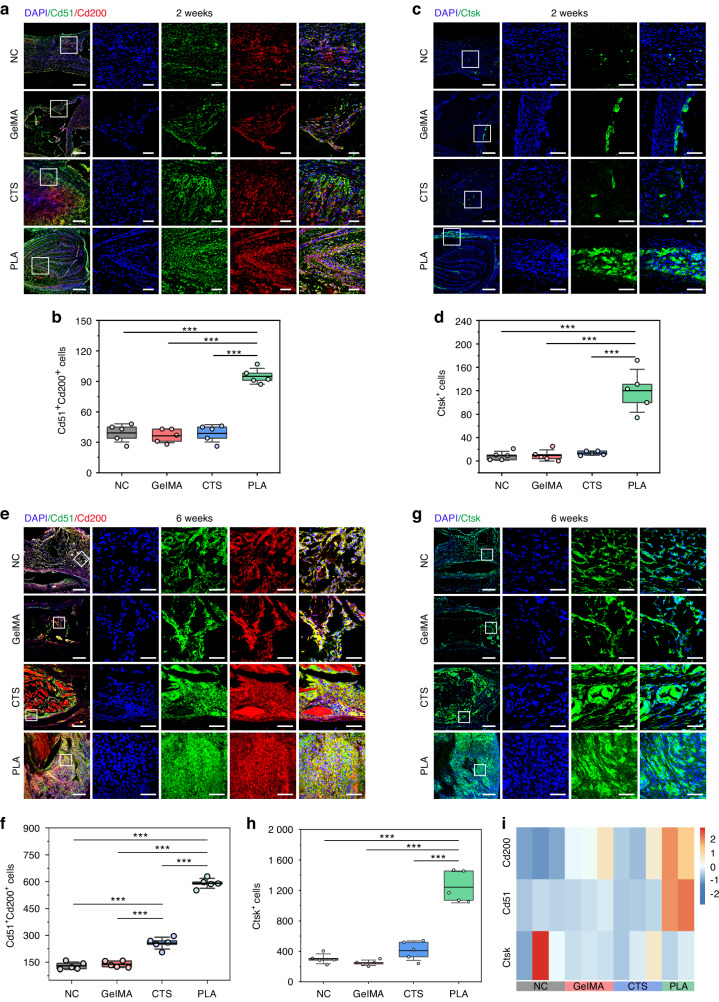
Cellular decoding on MSC identification in the newborn tissue. IF images showing the co-staining of skeletal stem and progenitor cells marker Cd51 (in green) and Cd200 (in red) in suture-bony composites at 2 weeks (**a**) and 6 weeks (**e**) post-surgery. **b**, **f** Statistical data for (**a**, **e**), respectively. IF images showing the periosteum-derived MSC marker Ctsk (in green) at 2 weeks (**c**) and 6 weeks (**g**) post-surgery. **d**, **h** Statistical data for (**c**, **g**). The high-magnification images are from the selected regions (white boxes) in the corresponding low-magnification images. Scar bar, 250 μm in low magnification and 50 μm in high magnification. **i** Transcriptome heatmap demonstrating the FPKM values of Cd200, Cd51, and Ctsk. NC, suture-bony composite defect without scaffold implantation. Data are expressed as mean ± SD. ***P < 0.001

**Fig. 6:**
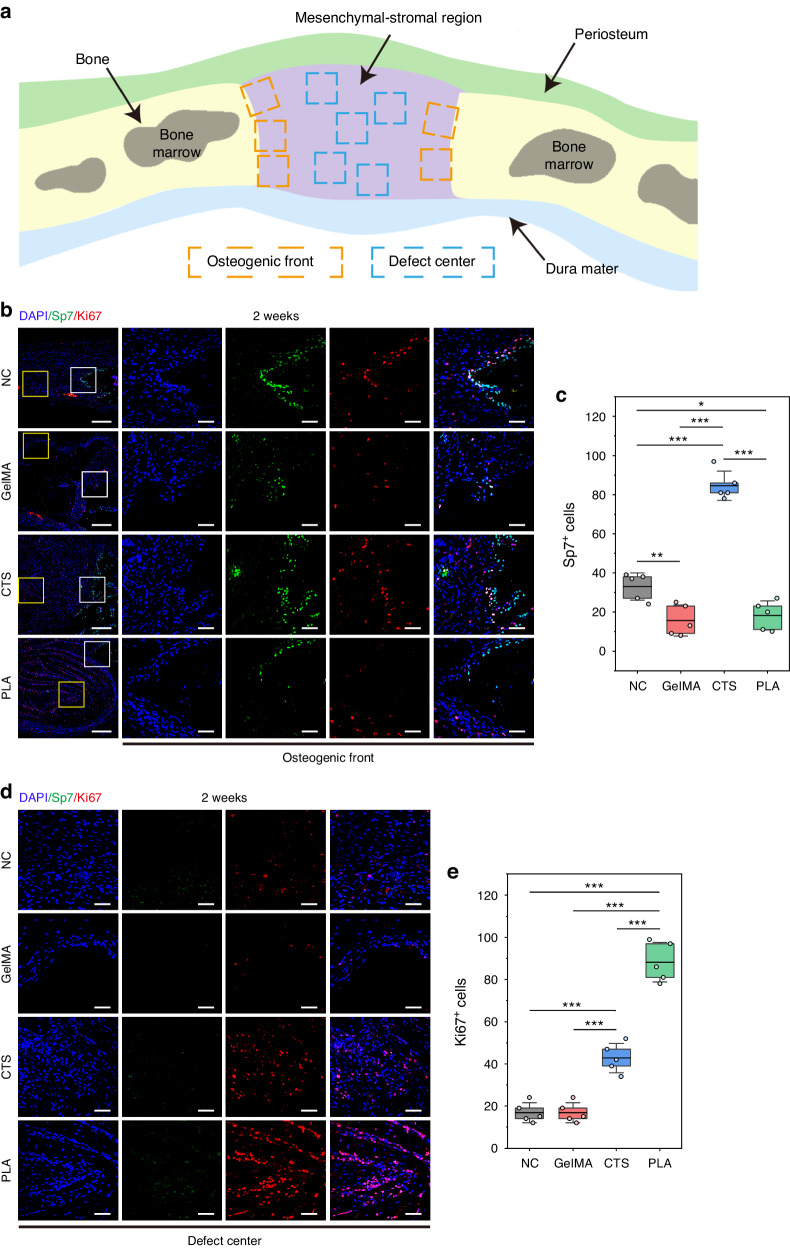
Cellular decoding on osteogenic lineage commitment in early stages. **a** Schematic representation of the random selection area at the osteogenic front or the defect center of suture-bony composite defect. **b** IF images displaying the co-staining of OB marker Sp7 (in green) and proliferation marker Ki67 (in red) at the osteogenic front 2 weeks post-surgery. The high-magnification images are from the selected regions (white boxes) in the low-magnification images. Scar bar, 250 μm in low magnification and 50 μm in high magnification. **c** Statistical data of Sp7^+^ cells at the osteogenic front (**b**). **d** IF images displaying the co-staining of Sp7 (in green) and Ki67 (in red) at the defect center 2 weeks post-surgery. The images are magnified from the selected regions (yellow boxes in **b**). Scar bar, 50 μm. **e** Statistical data of Ki67^+^ cells at the defect center (**d**). NC, suture-bony composite defect without scaffold implantation. Data are expressed as mean ± SD. *P < 0.05; **P < 0.01; ***P < 0.001

**Fig. 7:**
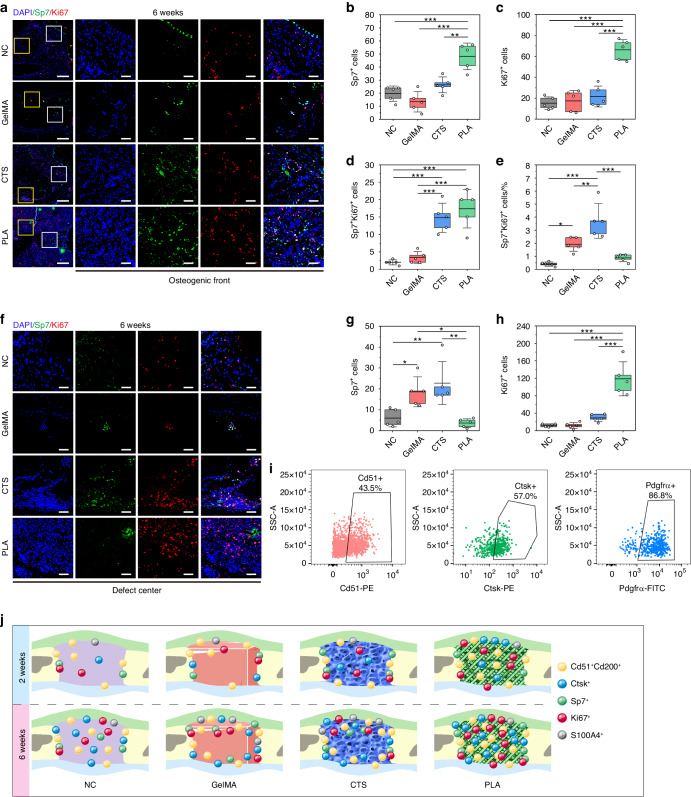
Cellular decoding on osteogenic lineage commitment in the later period. **a** IF images displaying the co-staining of OB marker Sp7 (in green) and proliferation marker Ki67 (in red) at the osteogenic front 6 weeks post-surgery. The high-magnification images are from the selected regions (white boxes) in the low-magnification images. Scar bar, 250 μm in low magnification and 50 μm in high magnification. Statistical data of Sp7^+^ cells (**b**), Ki67^+^ cells (**c**), Sp7^+^Ki67^+^ cells (**d**), and Sp7^+^Ki67^+^ cell proportion (**e**) at the osteogenic front (**a**). **f** IF images displaying the co-staining of Sp7 (in green) and Ki67 (in red) at the defect center 6 weeks post-surgery. The images are magnified from the selected regions (yellow boxes in **a**). Scar bar, 50 μm. Statistical data of Sp7^+^ cells (**g**) and Ki67^+^ cells (**h**) at the defect center (**f**). **i** FCM analysis of single-cell suspensions prepared from the suture-bony composite defects implanted with PLA for 6 weeks. **j** Schematic diagram summarizing the cellular composition within the suture-bony composite defects of NC, GelMA, CTS, and PLA at 2 weeks and 6 weeks post-surgery. NC, suture-bony composite defect without scaffold implantation. Data are expressed as mean ± SD. *P < 0.05; **P < 0.01; ***P < 0.001

Subsequently, we examined whether cells preserved within the defects expressed mesenchymal stem and progenitor cell markers. As depicted in Fig. 5, NC exhibited restricted MSC ingrowth and maintenance at both 2-week and 6-week time points. Additionally, the efficacy of GelMA and CTS in attracting MSC is not satisfactory either (Fig. 5). Whereas, PLA recruited a significantly higher number of Cd51^+^Cd200^+^ skeletal stem/progenitor cells 2 weeks post-operation (Fig. 5a, b). These cells migrated towards the defect center following the coiling direction of PLA and were also observed inside the spinning (Fig. 5a). By 6 weeks, PLA sustained a notably enlarged population of Cd51^+^Cd200^+^ cells, far from that of the other groups (Fig. 5e, f). Conforming to protein-level findings, transcriptomic analysis identified significant upregulation of *Cd51* and *Cd200* in PLA (Fig. 5i). Distinct from Cd51^+^Cd200^+^ cells, only a minority of MSC in the defects were derived from the periosteum at 2 weeks (Fig. 5c). Compared with other groups, PLA attracted a relatively higher number of Ctsk^+^ MSC at 2 weeks, predominantly located in the thickened periosteum, with some also migrating along PLA towards the defect center (Fig. 5c, d). When progressing to 6 weeks, abundant Ctsk^+^ MSC were present within the newborn tissue of PLA (Fig. 5g, h). Nonetheless, the genetic level of *Ctsk* in neonatal tissue did not increase simultaneously (Fig. 5i), which might be attributed to the conclusion of the high-expression phase of *Ctsk*. Given that the proliferation marker Ki67 was broadly expressed within the defects of PLA at both 2 weeks (Fig. 6d, e) and 6 weeks (Fig. 7a, c, f, h), the substantial existence of MSC at 6 weeks may arise from the rapid self-renewal of early ingrowth cells. To enhance the confirmation, single-cell suspensions were prepared from the newborn tissue of suture-bony composite defects implanted with PLA for 6 weeks. Flow cytometry (FCM) results showed that the digested cells comprised 43.5% Cd51^+^ cells, 57.0% Ctsk^+^ cells, and 86.8% Pdgfrα^+^ cells (Fig. 7i). Thus, it can be concluded that PLA promoted MSC ingrowth and self-renewal, contributing to the reconstruction of suture mesenchyme.

### Spatial-temporal decoding of osteogenic lineage commitment dynamics in PLA

Next, the osteogenic lineage commitment occurred at the osteogenic front or the defect center was detected separately referring to the region selection method illustrated in Fig. 6a. At the early time point, OB were solely detected at the osteogenic front of each group (Fig. 6b). Among them, CTS significantly promoted the osteogenic orientation at the repairing forefronts (Fig. 6b, c). Surprisingly, at the later time point, there were more Sp7^+^ cells (Fig. 7a, b) as well as Sp7^+^Ki67^+^ cells (Fig. 7a, d) at the osteogenic front of PLA, ensuring robust osseointegration between PLA and the bony edge and possibly contributing to the subsequent bone healing. Despite this, PLA demonstrated the lowest proportion of Sp7^+^Ki67^+^ cells (Fig. 7e), suggesting that most cells at the osteogenic front of PLA remained undifferentiated by 6 weeks. Meanwhile, PLA exhibited the lowest count of Sp7^+^ cells at the defect center (Fig. 7f, g), re-verifying its potential to restore suture mesenchyme and maintain its patency. All the cellular decoding outcomes were summarized in a schematic diagram (Fig. 7j), offering a comprehensive understanding of cellular composition within newborn tissue of suture-bony composite defects implanted with different scaffolds.

### Mechanistic validation of PLA’s cellular manipulation on suture-bony complex reconstruction in vitro

**Fig. 8:**
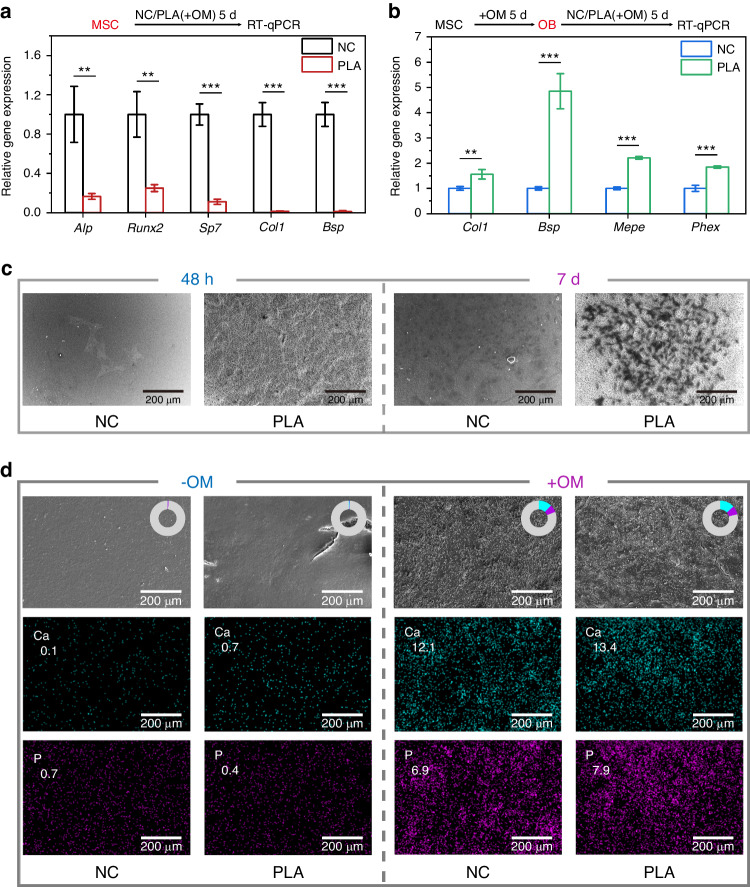
Mechanistic validation of PLA’s control of cell fate in vitro. RT-qPCR detecting osteogenic gene expressions in MSC (**a**) or OB (**b**). Each sample was examined in triplicate. *Gapdh* was used as the internal control. **c** SEM imaging capturing cell attachment at 48 h, followed by colony formation after 7 days. For colony formation detection, MSC were incubated in the complete medium. **d** EDS elemental mapping of calcium (Ca), phosphorus (P), carbon (C), oxygen (O), and nitrogen (N) in the corresponding SEM images of MSC treated with (+OM) or without (-OM) osteogenic medium. The pie charts and the numbers in the images display the elemental proportions. The gray region represented the sum of C, O, and N elements. NC, MSC seeded on glass slides or in blank wells. PLA, MSC seeded on PLA. Data are expressed as mean ± SD. **P < 0.01; ***P < 0.001

In vivo, PLA exhibited spatiotemporal regulation of osteogenic lineage commitment, with strong edge osteogenesis but minimal central ossification in suture-bony composite defects (Fig. 7). Correspondingly, we established two models in vitro (Fig. 8). First, we simulated PLA’s effects at the defect center by seeding undifferentiated MSC onto the scaffold surface. Genetic analysis revealed that PLA impeded the osteogenic differentiation of MSC with significantly lower levels of *Alp*, *Runx2*, *Sp7*, *Col1*, and *Bsp* (Fig. 8a). Besides, following 7 days of culture, a limited number of MSC on PLA developed into sizable cell colonies by SEM (Fig. 8c), suggesting that MSC on PLA could replicate and self-renew in the absence of osteogenic induction (Fig. 8c). Hence, PLA sustained MSC self-renewal and inhibited their osteogenic differentiation, with the presence of abundant proliferating MSC (Fig. 5) and limited OB (Fig. 7) observed at the defect center in vivo. To verify the osteogenic-inductive properties of PLA at the osteogenic front, a parallel model was adopted. Briefly, MSC were cultured in osteogenic medium (OM) for 5 days to generate OB (Fig. S7) and subsequently seeded onto PLA. As shown in Fig. 8b, PLA promoted the expression of middle/late osteogenic genes (*Col1*, *Bsp*, *Mepe*, and *Phex*) in OB. That is to say, for differentiated cells, PLA maintained and even augmented their osteogenic orientation (Fig. 8b). Collectively, in the potent and persistent osteoinductive environment in vitro, PLA demonstrated cell-specific actions on MSC and OB. As for the relevant in vivo setting, osteogenic signals are concentrated at the osteogenic front and attenuated at the defect center, accounting for the spatiotemporal effects of PLA.

By prolonging osteogenic induction in vitro, MSC on PLA exhibited the capacity to form mineralized nodules (Fig. 8d). SEM images revealed comparable rough surfaces of mineralized nodules on glass slides (NC) and PLA, with EDS mapping indicating similar Ca and P deposition (Fig. 8d). Persistent osteogenic induction in vitro prompts partial MSC to adopt osteogenic commitment, thereby contributing to the formation of mineralized nodules. Similarly, osteogenic signals at the osteogenic front ensure the proper osteointegration. As the reconstruction of suture-bony composite defects proceeds, the signals gradually decline to a basal level, leading to slower hard tissue restoration and the formation of regenerated sutures by residual nascent mesenchyme.

## Discussion

Due to increased trauma, infections, malignancies, and neurological diseases, the prevalence of calvarial defects rises rapidly.^38^ Yearly, approximately 69 million individuals suffer from traumatic head injuries worldwide.^39^ In 2020, the global cranial implants market was valued at 1.05 billion USD, which is predicted to reach 1.77 billion USD by 2028.^38^ However, despite substantial clinical demands and extensive economic burdens, current clinical strategies often fall short of restoring cranial mesenchyme, leading to unfavorable outcomes for calvarial defect management. In pediatric cases, the consequences of suture loss are even worse. Cranial sutures function as the main growth centers in the skull during periods of development.^1,40,41^ The absence of these structures, due to postnatal cranial defects or congenital craniosynostosis, disrupts skull-brain growth balance, limiting brain expansion and giving rise to various problems, such as increased intracranial pressure, hydrocephalus, cognitive issues, blindness, dyspnea, and epilepsy.^1,42^ Moreover, following surgical interventions, a substantial portion of pediatric patients necessitate secondary surgeries owing to a recurrence of cranial suture closure.^21,43^ Therefore, given the lack of direct exploration in the current research, investigating cranial suture regeneration in adolescents emerges as a vital focus for our future studies.

Through the calvarial suture-bony composite defect model established in this research, our findings revealed a distinct difficulty in cell ingrowth and recruitment without scaffold implantation (Fig. 7j). Despite reduced fibrosis risk (Fig. 4e, f), NC displayed an inability to attract functional cells including Cd51^+^Cd200^+^ and Ctsk^+^ MSC, Sp7^+^ OB, and proliferating Ki67^+^ cells (Fig. 7j). Attracting cells to the defect center is a crucial step in tissue engineering.^44^ Especially in critical-sized bone defects, the distance between the osteogenic front and defect center is detrimental to the delivery of nutrients, metabolites, osteogenic molecules, and cells, thus impeding bone healing.^44^ Nonetheless, NC did not end in vacuolation, bone malunion, or non-union due to the lack of cell recruitment. Conversely, we observed a complete bony union with a fused cranial suture 6 months post-operation (Fig. S4). The occurrence of hard tissue restoration is attributed to the innate self-regeneration capacity of bone tissue, given the absence of critical-sized defects in our experimental setup.^45^ However, under the conditions of natural healing, there exists an inherent deficiency in attracting MSC ingrowth and preserving their self-renewal. The strong osteogenic restoration coupled with inadequate mesenchymal regeneration leads to suture closure. This highlights the importance of cell fate control in suture-bony complex regeneration.^8,26^

The biophysical effects of materials, such as their compositional/degradable dynamics, mechanical properties, 2D topography, and 3D geometry, profoundly affect cell behaviors.^46^ Scaffold pore size, particularly, has a notable impact on MSC and OB,^47–55^ with small pores (diameter <125 μm) inhibiting MSC differentiation by restricting vasculature and promoting immature matrix.^47^ Conversely, large pores (diameter >250 μm) support sufficient osteogenic niches and robust vascularization for bone formation.^47^ In contrast to non-porous GelMA and macroporous CTS, the dense packing and small pores of PLA potentially enhance MSC ingrowth and maintain their stemness, while preserving OB at the bony edge. As another critical concern, degradability contributes to the constant renewal of the scaffold surface.^56^ It is reported that dynamic substrates may be essential for the scaffold capable of regulating the spatiotemporal interactions among various cell populations.^57^ Moreover, as the scaffold degrades, the pore size and interconnectivity increase, enhancing the delivery of cells and growth factors for subsequent tissue repair such as bone elongation by OB along the osteogenic front.^58,59^ Despite non-degradability in vitro (Fig. S1b), PLA displayed superior degradation performance in vivo (Fig. 2f, g). As for poorly degradable GelMA and CTS, the adhesive and proliferated cells on the surface restrict cell penetration to the scaffold center, possibly leading to a necrotic core over time.^60^ Meanwhile, large amounts of extracellular matrix accumulate around the scaffold, resulting in the adverse outcome of fibrosis (Fig. 4e, f). However, due to the distinct characteristics of GelMA, CTS, and PLA, featuring variations in polymer chain structure, chemical moieties, molecular weight, and other properties, it remains inconclusive to attribute the observed differences in biological functionality to specific material properties. In the subsequent investigations, our emphasis will be directed towards the screening out PLA, wherein we will systematically adjust its single parameter and precisely elucidate the material characteristics influencing mesenchymal regeneration.

As revealed by our transcriptome data, PLA is instrumental in establishing a microenvironment promoting cellular interaction via chemokines and cytokines. Particularly, the presence of PLA scaffolds significantly boosts macrophage metabolic activity, evidenced by a surge in cytokines such as IL-6, IL-8, and MCP-1.^61^ This specific macrophage secretion pattern, triggered by PLA, is closely linked to the increased MSC recruitment,^61^ shedding light on the mechanisms facilitating mesenchymal tissue regeneration. Besides, suture fate determination is intricately governed by various signaling pathways such as cWnt (canonical Wnt/β-catenin), FGF (fibroblast growth factor), BMP (bone morphogenetic protein), and IHH (Indian hedgehog) signaling pathways.^3,9,62^ The activation of the cWnt pathway inhibits posterior frontal suture closure but promotes sagittal suture closure through modification of endochondral ossification in mice.^62^ Besides, the cWnt pathway indirectly affects MSC lineage commitment by modulating the equilibrium between FGF and BMP pathways.^63^ β-catenin activation, followed by FGF disruption and BMP activation, leads to chondrogenic differentiation of suture-derived MSC, thus driving suture fusion via ectopic chondrogenesis and endochondral ossification.^63^ Additional researchers have documented the bifunctional role of the cWnt pathway in osteogenic lineage commitment, inhibiting osteogenesis in undifferentiated MSC and juvenile calvarial OB while promoting it in mature OB.^64^ Similarly, PLA hindered the osteogenic differentiation of MSC but enhanced it in OB. BMP receptor type 1 A (BMPR1A) maintains MSC properties during craniofacial development.^65^ Interestingly, suture fusion caused by BMPR1A loss manifests as abnormal ossification deriving from the suture mesenchyme and moving toward the osteogenic fronts.^65^ Likewise, the presence of diffuse ossification was also observed at the defect center of NC, GelMA, and CTS (Fig. 2g). Moreover, the interplay between MSC and osteoclasts mediated by BMP-IHH signaling also contributes to suture homeostasis.^9^ BMP signaling induces osteoprogenitor cells to secrete IHH, which subsequently promotes the osteogenic differentiation of MSC. Meanwhile, BMP-mediated IHH signaling functions synergistically with nuclear factor kappa-B ligand (RANKL) to promote differentiation and resorption activity of osteoclasts.^9^ According to the transcriptome data, GSEA analysis suggested that PLA significantly activated NF-κB and HIF-1 signaling pathways in comparison with the other three groups (Fig. S5f, g). However, the present studies lack conclusive evidence directly linking these two pathways with cranial sutures. Our follow-up experiments will center on elucidating the molecular mechanisms underlying PLA’s well-orchestrated cell fate determination. We aim to provide insights into effective bone-healing pathways and possible scaffold-related drawbacks, thereby offering novel strategies for optimizing specific scaffolds.^66^

In this study, three most commonly used tissue engineering scaffolds with distinct properties were selected and transplanted into calvarial suture-bony composite defects. A thorough investigation was conducted to compare their transcriptional regulation and influence on cell behaviors in terms of cell adhesion, intrinsic recruitment, stemness maintenance, cellular renewal, and osteogenic differentiation. Our findings indicated that in the absence of scaffold implantation, namely the natural healing group after defects, ingrowth and amplification of self-renewable MSC into the defect sites were very difficult. Being transplanted with inappropriate scaffolds, like GelMA and CTS, excessive fibrogenesis and ectopic ossification were observed at the defect center, suggesting the possibility of adverse suture closure in the future. Conversely, only PLA was demonstrated to facilitate MSC ingrowth and self-renewal within the central regions and sustained osteointegration/osteogenesis at the repairing forefronts. Therefore, PLA was identified as a suitable scaffold for suture mesenchyme reconstruction. Our findings, for the first time, decoded the cellular fate controls by scaffolds in calvarial suture-bony composite defects, offering fundamental insights into regenerating mesenchymal suture-like structures to avoid craniosynostosis.

## Materials and methods

### Ethical statements

All experimental designs and procedures in this study were reviewed and approved by the Ethical Committees of West China School of Stomatology, Sichuan University (WCHSIRB-D-2021-597).

### Preparation of GelMA, CTS, and PLA

To synthesize GelMA, 10% (w/v) type A porcine skin gelatin was dissolved in PBS at 60 °C, followed by the dropwise addition of 1.25% (v/v) methacrylic anhydride. This reaction was stopped by adding double PBS. Then, the solution was dialyzed using 12–14 kD dialysis tubing in distilled water for 1 week. After freezing at −20 °C overnight and lyophilizing for 72 h, sponge GelMA was obtained. 20% (w/v) GelMA was dissolved in PBS with 0.1% (w/v) photoinitiator LAP (Lithium Phenyl-2,4,6-trimethylbenzoylphosphinate). The mixture was exposed to ultraviolet light (6.9 W/cm^2^, 360–489 nm) for 2 min at room temperature to obtain GelMA hydrogels. As for CTS, 0.1 g CTS was dissolved in 10 mL of 2 wt% acetic acid solution. Subsequently, the crosslinking agent tripolyphosphate was added to the CTS solution. Thorough mixing of the solution took place at room temperature. The resulting hydrogels underwent extensive washing with distilled water to eliminate unreacted monomers. Finally, the hydrogels were subjected to freeze-drying for preservation. As for PLA, poly(L-lactide) (PLA, M_n_ = 9.1 × 10^4^ g/mol, M_w_ = 2.1 × 10^5^ g/mol, M_w_/M_n_ = 2.3) were kindly provided by Zhejiang Hisun Biomaterials Co. Ltd. (China). A homogeneous solution was prepared by dissolving PLA in a mixture of dichloromethane and N, N-dimethylformamide with a volume ratio of 7:3. A total of 5 mL of dissolved solution was loaded into a syringe for electrostatic spinning. The solution was pushed at a rate of 0.8 mL/h under a voltage of 18 kV for 8 h, resulting in the formation of a solid PLA membrane. Finally, the fibers were vacuum-dried at 50 °C for 24 h to remove residual solvent.

### Characterizations

The morphological imaging and elemental analysis were performed by a JOEL JSM-5900LV field-emission SEM (Japan) equipped with EDS (Ultim Extreme, Oxford Instruments, England). Rheological measurements were conducted with a rotational rheometer (AR2000EX, TA instruments, USA) to evaluate the mechanical responses of the scaffolds. Relative modulus was calculated using the Eq. (1). The scaffold with the highest modulus was set as the control. Equation (2) was utilized to assess relative structural stability. The curvature of cycle scan curves for each group was determined and the most structurally stable material was set as the control. TGA was conducted using a thermogravimetric analyzer (Q600, TA Instruments, USA). The decomposition temperature of the samples was determined by heating them from 30 to 800 °C at a rate of 10 °C/min under a nitrogen atmosphere. Degradation tests were carried out in PBS at 37 °C in vitro. We combined TGA and in vitro degradation results to assess thermal stability. Briefly, a decrease in weight within 100 °C on TGA deducted 30 points from the 100-point thermal stability score. Meanwhile, weight loss in the 8-week degradation test resulted in another 30-point deduction.

1 $$({\rm{Relative}})\,{\rm{Modulus}}( \% )=\frac{\log {Modulu}{s}_{{sample}}}{\begin{array}{c}\log {Modulu}{s}_{{control}}\\ \,\end{array}}\times 100 \%$$

2 $$({\rm{Relative}})\,{\rm{Structual}}\; {\rm{stability}}( \% )=\frac{\left|{{\rm{Curvature}}}_{{\rm{sample}}}\right|}{\left|{{\rm{Curvature}}}_{{\rm{Control}}}\right|}\times 100 \%$$

### Isolation and culture of MSC

Periosteal MSC were obtained from 10-week-old SD rats by subjecting the femoral and tibial periosteum to enzymatic digestion using type 1 collagenase (3.5 mg/mL) at 37 °C for 1 h. The cells were cultured and expanded in the complete medium (α-MEM supplemented with 10% fetal bovine serum (FBS) and 1% penicillin-streptomycin) at 37 °C in a humidified atmosphere containing 5% CO_2_. The culture medium was refreshed every 2 days. The cells were passaged when they reached 80% confluence. Passages 1–2 MSC were utilized for the in vitro studies.

### Cell experiments in vitro

Following varying degrees of water swelling, the scaffolds were shaped into 1.4–1.5 cm diameter cylinders or discs (Fig. 1b) to cover the bottom of 24-well microtiter plates. MSC were seeded on the scaffolds. The cellular morphology of MSC on sterile glass slides, GelMA, CTS, and PLA was observed by SEM. The cell adhesion and proliferation were evaluated using CCK-8 Cell Proliferation and Cytotoxicity Assay Kit following the manufacturer’s manual. Briefly, MSC were seeded on the scaffolds at a density of 2 × 10^5^ per well. The absorbance of the reaction solution was determined using a microtiter plate spectrophotometer (Multiskan GO; Thermo Scientific, Waltham, MA, USA) after MSC were seeded for 6 h and 1, 3, and 5 days. OD_490_ minus OD_630_ of 6 h represented the number of adhesive living cells on the scaffold. OD_490_ minus OD_630_ of 1, 3, and 5 days represented the number of proliferated living cells. Relative cell adhesion was calculated according to Eq. (3) based on the 6-h measurements. For cell survival, MSC co-cultured with various scaffolds were collected by trypsin digestion and stained by Annexin V-PE/7-AAD Apoptosis Detection Kit. FCM was used to estimate the percentage of live (Annexin V^-^/7-AAD^-^), early apoptotic (Annexin V^+^/7-AAD^-^), late apoptotic (Annexin V^+^/7-AAD^+^), and necrotic (7-AAD^+^) cells following 72 h of treatments. The ability of cells to form colonies was evaluated using a colony formation assay. MSC were cultured on glass slides or PLA at the bottom of 24-well microtiter plates with 50 cells per well. At 48 h and 7 days after incubation, the cells were fixed with 4% paraformaldehyde for 30 min, followed by dehydration using graded ethanol series (30%, 50%, 75%, 85%, 95%, and 100%; 15 min each). Cell colonies were monitored by SEM. For the above experiments, the negative control (NC) referred to the group where cells were cultured on sterile glass slides or in empty wells.

3 $$({\rm{Relative}}){\rm{Cell\; adhesion}}( \% )=\frac{{\left(O{D}_{490}-O{D}_{630}\right)}_{{sample}{of}6h}}{{\left(O{D}_{490}-O{D}_{630}\right)}_{{NC}{of}6h}}\times 100 \%$$

### Osteogenic induction in vitro

OM referred to α-MEM added with 10 nM dexamethasone, 10 mM glycerophosphate, 50 g/mL L-ascorbic acid, 5% FBS, and 1% penicillin-streptomycin. MSC on sterile glass slides (NC) or PLA were incubated in OM for 14 days. Then, SEM equipped with EDS was performed to detect C, O, N, calcium (Ca), and phosphorus (P) elements deposited on the scaffolds. For RT-qPCR, MSC were treated with OM for 5 days to obtain OB (Fig. S7). MSC or OB were inoculated on PLA in OM for 5 days before RNA extraction. Cells cultured in empty wells were set as the negative control (NC). TRIzol was employed to extract the total RNA. NanoDrop 2000 (Thermo Scientific) served to assess and measure the quality and quantity of RNA. The cDNA was synthesized using HiScript III RT SuperMix for qPCR (+gDNA wiper). The instructions of the ChamQ Universal SYBR qPCR Master Mix were followed to prepare the PCR reagent mixture. Then, via the suggested method, RT-qPCR was performed in triplicate for each sample using a Real-Time PCR System (C1000 Thermal Cycler; Bio-Rad, Hercules, CA, USA). The tested osteogenic genes and the corresponding sequences of PCR primers are listed in Table S1. Glyceraldehyde-3-phosphate dehydrogenase (*Gadph*) was set as the reference gene. The fold change of gene expression was calculated using the ΔΔCT method.

### Animal experiments

Male SD rats (300 g) were obtained from Byrness Weil Biotech Ltd (Chengdu, China). Under general anesthesia, a midline sagittal incision was made on the calvaria to fully expose the bilateral coronal sutures. The overlying periosteum was carefully removed using a curette. A micro dental drill (diameter=1.2 mm) was then used to create 2 mm by 4 mm rectangular defects across coronal sutures. Different scaffolds with proper size were implanted into the defects. The scalp was closed using 5.0 polyglactin stitches. The rats were sacrificed at 2 weeks or 6 weeks after surgery.

### Biosafety evaluation in vivo

At 6 weeks post-surgery, measurements were taken for rat body weight, head length, head width, cranial length, and cranial width. Head length was determined by the distance from the nose tip to the cervical spine joint, while head width was assessed by the ear base distance. Moreover, Mimics 20.0 software enabled separate evaluations of cranial length and width, achieved by measuring the straight-line distance between the start and end points of the sagittal suture and coronal suture based on CT scans. Meanwhile, major organs (kidneys, livers, lungs, hearts, and spleens) were extracted for histological examination through Hematoxylin and Eosin (H&E) staining. Blood samples were collected from rat abdominal aortas for hematological and biochemical analysis. By combining in vivo and in vitro biocompatibility evaluation results, we established a scaffold biocompatibility rating using Eq. (4).

4 $$\begin{array}{l}{\rm{Biompatibility}}=100-20\times {\rm{n}}\,\left(\right.{\rm{the}}\; {\rm{count}}\; {\rm{of}}\; {\rm{experiments}}\; \\{\rm{indicating}}\;{\rm{worse}}\; {\rm{biocompatibility}}\; {\rm{than}}\; {\rm{NC}}\left.\right)\end{array}$$

### µCT evaluation

6-week calvarial samples were scanned using µCT (Scanco Medical AG, Bassersdorf, Switzerland). The scan parameters were as follows: X-ray tube potential, 70 kVp; X-ray intensity, 0.2 mA; filter, AL 0.5 mm; integration time, 1 × 300 ms; and voxel size, 10 μm. The 3D images were reconstructed by Scanco medical visualizer software. Dataviewer and Ctan software were used to acquire residual suture volume (RSV). Mimics 20.0 software was used to obtain cross-sectional images. The degree of defect closure (%) was calculated by Eq. (5). The control group (Ctr) received no scaffold implantation in the defects. Positive values signify the promotion of suture-bony composite defect closure, while negative values indicate the inhibition of suture-bony composite defect closure.

5 $${\rm{Defect}}\; {\rm{closure}}\,( \% )=\frac{\left({{\rm{RSV}}}_{{\rm{sample}}}-{\rm{RSV}}{\rm{Ctr}}\right)}{{\rm{RSV}}{\rm{Ctr}}}\times 100 \%$$

### Histological evaluation

The harvested cranial bones were fixed in 4% paraformaldehyde for 24 h and decalcified in 12% EDTA (w/v, pH=7.2) for 6 weeks. Then, the samples were dehydrated, embedded in paraffin, and sectioned at 6 μm. H&E and Masson’s trichrome staining were applied for histological analysis following the product manual. Fiji software was employed to quantitatively analyze blue-stained regenerated hard tissues in 10 randomly selected regions (5 from the osteogenic front and 5 from the defect center). Random region selections followed the guidance in Fig. 6a.

### RNA-seq analysis

6 weeks post-surgery, nascent tissue from the suture-bony composite defects, implanted with GelMA, CTS, and PLA, or without any scaffold (NC), was isolated and subjected to digestion in type 1 collagenase (3.5 mg/mL) at 37 °C for 1 h. Subsequently, tissue debris was removed. The digested cells were collected by centrifugation (500 g, 10 min) and then incubated in Red Cell Lysis Buffer at room temperature for 2 min. Following this, the cells were fixed and permeabilized using BD Cytofix/Cytoperm^TM^ Fixation/Permeabilization kit at 4 °C for 10 min. After another centrifugation (500 g, 5 min), the cells were incubated with CD45 (clone 30-F11) Brilliant Violet 510 (Table S2) at room temperature for 30 min. The supernatant was discarded. The cells were washed twice in BD Perm/Wash™ Buffer, resuspended in PBS, and stored at 4 °C in the dark. Cd45^-^ cells were sorted (Fig. 3a) by FACSAria SORP cytometer (BD Biosciences, San Jose, CA, USA) for further transcriptome analysis. The total RNA extraction, RNA-seq, and bioinformatic data analysis were conducted by OE Biotech Co., Ltd. (Shanghai, China). The accession number for the RNA-seq data reported in this paper is GSE249260.

### IF staining

Paraffin slides were deparaffinized and antigen was retrieved with sodium citrate for 40 min at 95 °C. Then, tissue sections were permeabilized with 0.5% Triton-100 in PBS (PBST) for 15 min, blocked with 5% BSA in PBST for 20 min, and incubated with corresponding first antibodies (Table S2) at 4 °C overnight. On the next day, the sections were treated with particular second antibodies (Table S2) and DAPI (1:50) for 2 h at room temperature. A confocal laser scanning microscope (CLSM, FV3000, Olympus, Japan) was used to capture the images. Each sample was scanned at 5 random locations. The number of single- and double-positive cells was calculated using Fiji software. Cell proportion was calculated by using the amount of DAPI as the total cell number.

### FCM

A single-cell suspension was prepared from the nascent tissue of the suture-bony composite defects implanted with PLA for 6 weeks. The cells were incubated with the corresponding primary antibodies (Table S2) at room temperature for 30 min. The supernatant was discarded. The cells were washed twice in BD Perm/Wash™ Buffer, and subsequently incubated in the appropriate second antibodies (Table S2) at room temperature for 10 min. After another 2 rounds of washing, the samples were resuspended in PBS and stored at 4 °C in the dark. A FACSAria SORP cytometer (BD Biosciences, San Jose, CA, USA) was used for sample processing. The proportion of Cd51^+^, Pdgfrα^+^, and Ctsk^+^ cells among Cd45^-^ cells were analyzed using Flowjo v10.8.1. In vitro-expanded MSC were subjected to the same processing steps to formulate FCM gating strategies (Fig. S6).

### Statistical analysis

A minimum of three repetitions were performed for each experiment. Data were presented as mean ± standard deviation (SD). The statistical analysis was performed using IBM SPSS Statistics for Windows (Version 20.0, Released 2011; IBM, Armonk, NY, USA). Statistical analyses comparing the two groups were conducted using a t test. Differences among three or more groups were assessed through one-way ANOVA followed by Dunnett’s multiple comparisons test. Statistical significance was defined as *P* < 0.05. All graphical illustrations were obtained via Origin 8.0 software.

### Supplementary information

Supporting Information

## Data Availability

The accession number for the RNA-seq data reported in this paper is GSE249260. The data that support the findings of this study are available from the corresponding author upon reasonable request. All the original data and images are available from Fanyuan Yu upon request (fanyuan_yu@outlook.com).
