# Supplementary material for: Transcriptomic and cellular decoding of scaffolds-induced suture mesenchyme regeneration
Source: Int J Oral Sci. 2024 Apr 23;16:33. doi: 10.1038/s41368-024-00295-y (PMC11039626; doi:10.1038/s41368-024-00295-y)
Supplement: Supplementary file 1 — Supporting Information [file 41368_2024_295_MOESM1_ESM.pdf]

Supporting Information

Transcriptomic and Cellular Decoding of Scaffolds-induced Suture Mesenchyme Regeneration

Running title: Scaffold-induced Suture Mesenchyme Regeneration

Jiayi Wu[1, 2, *], Feifei Li[1,3, *], Peng Yu[1], Changhao Yu[1, 2], Chuyi Han[1], Yitian Wang[1], Fanyuan Yu[1, 2, #], Ling Ye[1, 2, #]

[1] State Key Laboratory of Oral Diseases & National Center for Stomatology & National Clinical Research Center for Oral Diseases, West China Hospital of Stomatology, Sichuan University, Chengdu, Sichuan, China

[2] Department of Endodontics, West China Hospital of Stomatology, Sichuan University, Chengdu, China

[3] Department of Pediatric Dentistry, West China Hospital of Stomatology, Sichuan University, Chengdu, China

* These authors contribute equally.

* Corresponding Authors:

E-mail: fanyuan_yu@outlook.com; yeling@scu.edu.cn

This file includes:

Supplementary Figures and Tables

Supplemental Figures

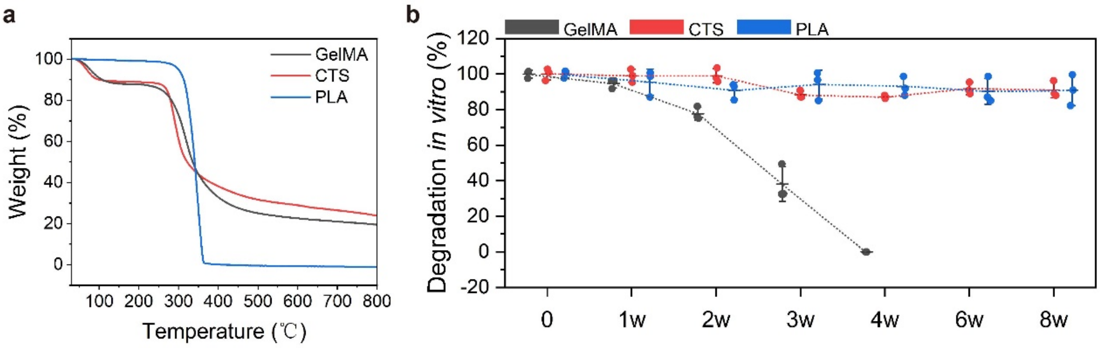

Supplementary Fig. 1 Thermal characteristics of the candidate scaffolds. **a** Thermal degradation performances of GelMA, CTS, and PLA characterized by TGA. **b** Degradation properties of GelMA, CTS, and PLA in PBS at 37°C in 8 weeks. Data are expressed as mean ± SD.

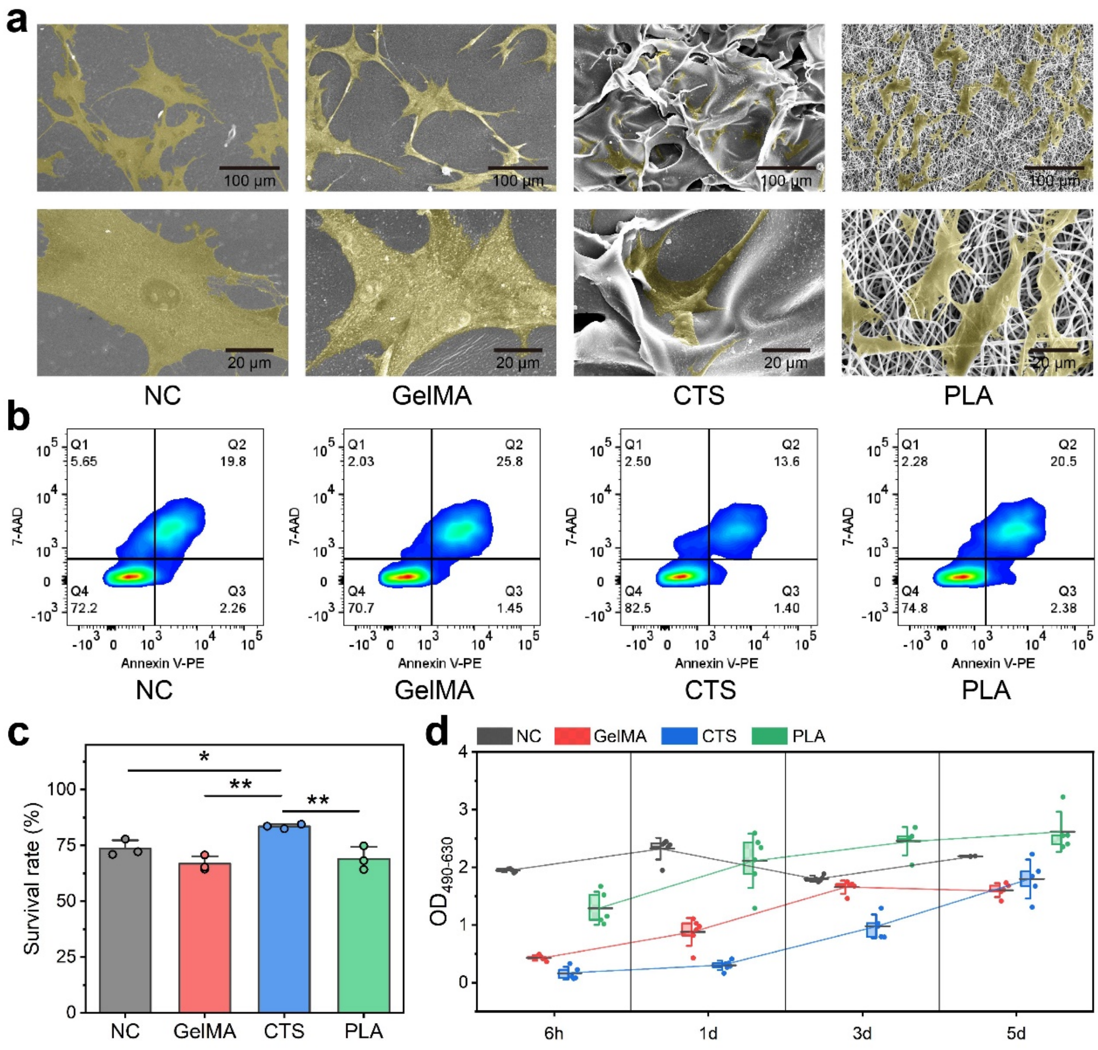

Supplementary Fig. 2 *In vitro* cytocompatibility of the scaffolds. **a** Cellular morphology of MSC (in yellow) on sterile glass slides (NC), GelMA, CTS, and PLA by SEM. **b** Representative FCM experiment depicting Annexin V^{+}/7-AAD^{-} (early apoptotic), Annexin V^{+}/7-AAD^{+} (late apoptotic), 7-AAD^{+} (necrotic), and Annexin V^{-}/7-AAD^{-} (survival) cells in each group. **c** Cell survival rate according to FCM (**b**). **d** CCK-8 results of MSC seeded on sterile glass slides (NC), GelMA, CTS, and PLA. OD_{490} minus OD_{630} of 6 hours represented the number of adhesive living cells on the scaffolds. OD_{490} minus OD_{630} of 1, 3, and 5 days represented the number of proliferated living cells. Data are expressed as mean ± SD. *p<0.05; **p<0.01.

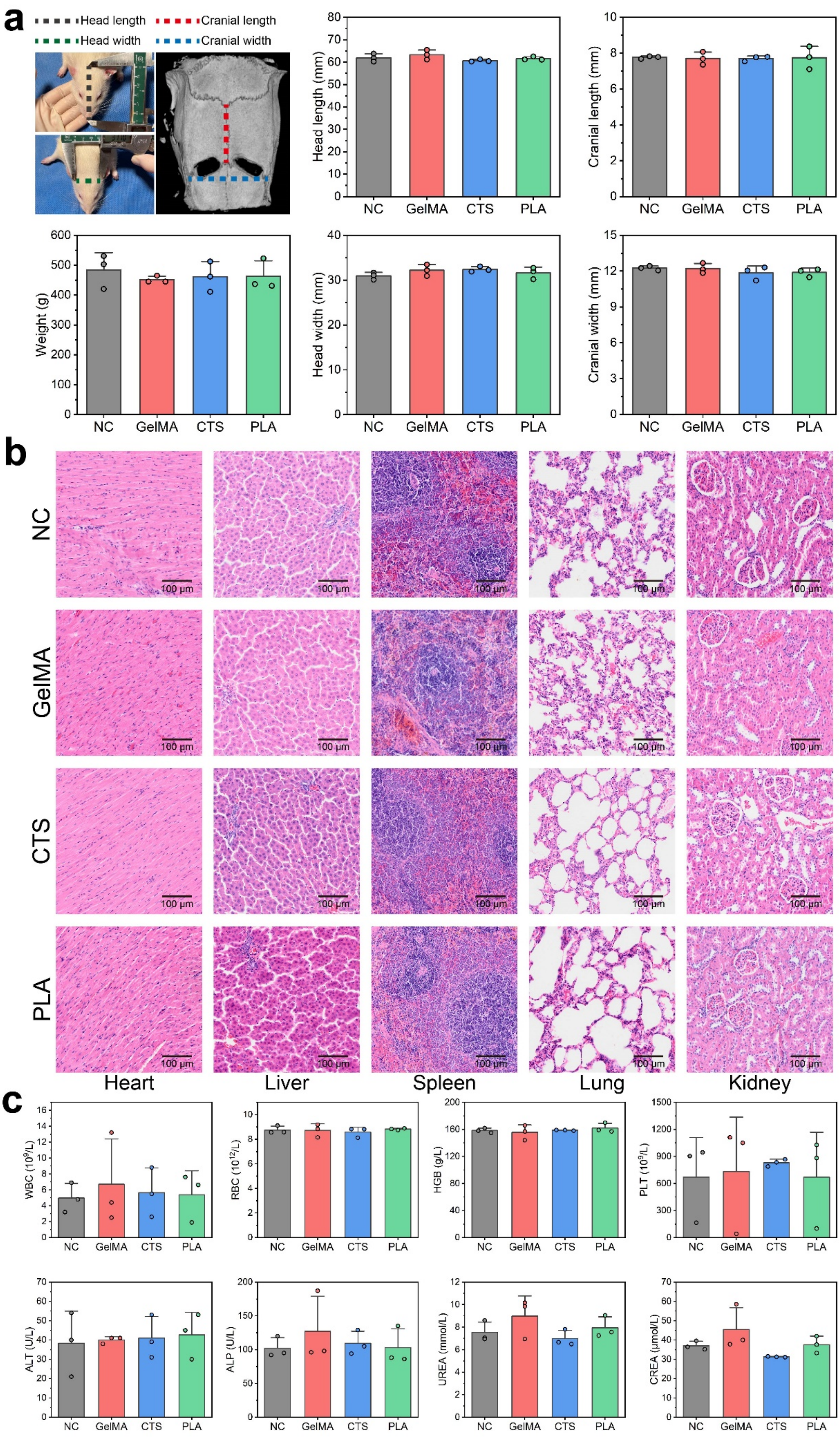

a
Head length
Cranial length
Head width
Cranial width
Head length (mm)
Cranial length (mm)
Weight (g)
Head width (mm)
Cranial width (mm)
NC
GelMA
CTS
PLA
b
NC
GelMA
CTS
PLA
100 μm
Heart
Liver
Spleen
Lung
Kidney
c
WBC (10^9/L)
RBC (10^{12}/L)
HGB (g/L)
PLT (10^9/L)
ALT (U/L)
ALP (U/L)
UREA (mmol/L)
CREA (μmol/L)

Supplementary Fig. 3 *In vivo* biosafety of the scaffolds. **a** Rat body weight, head length, head width, cranial length, and cranial width measured 6 weeks post-surgery. **b** Histological images of major organs (heart, liver, spleen, lung, and kidney) by H&E staining. **c** Hematological examination of white blood cells (WBC), red blood cells (RBC), hemoglobin (HGB), and platelets (PLT), alongside biochemical analysis of alanine aminotransferase (ALT), alkaline phosphatase (ALP), blood urea nitrogen (UREA), and creatinine (CREA). NC, suture-bony composite defects without scaffold implantation. Data are expressed as mean ± SD.

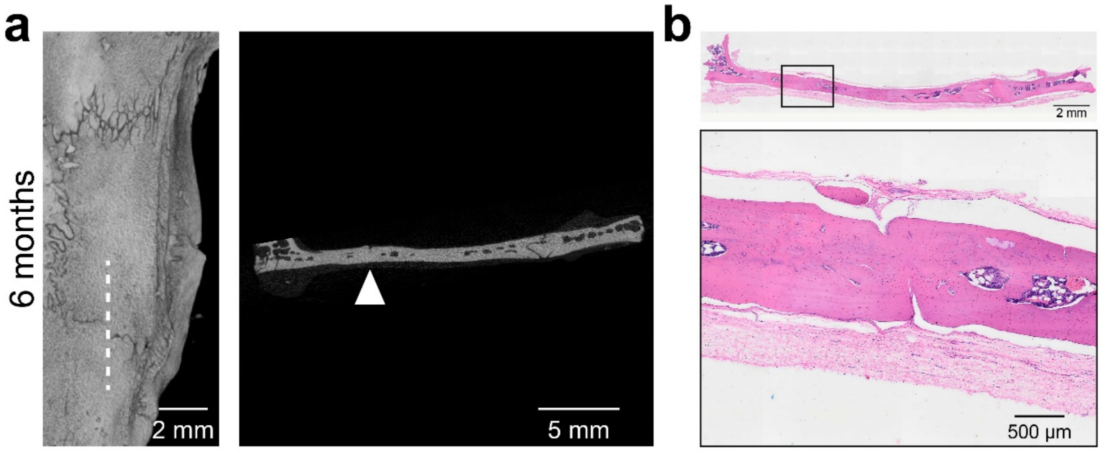

Supplementary Fig. 4 Long-term prognosis of calvarial suture-bony composite defects without scaffold implantation. **a** Representative μCT image and cross-sectional view of suture-bony composite defect without scaffold implantation 6 months postoperatively. The cross-section depicts the location indicated by the white dashed line in the 3D image. The white solid triangle indicates the location of the fused coronal suture. **b** H&E staining of (**a**). The high-magnification image demonstrating the fused coronal suture is from the selected region (black box) in the low-magnification image.

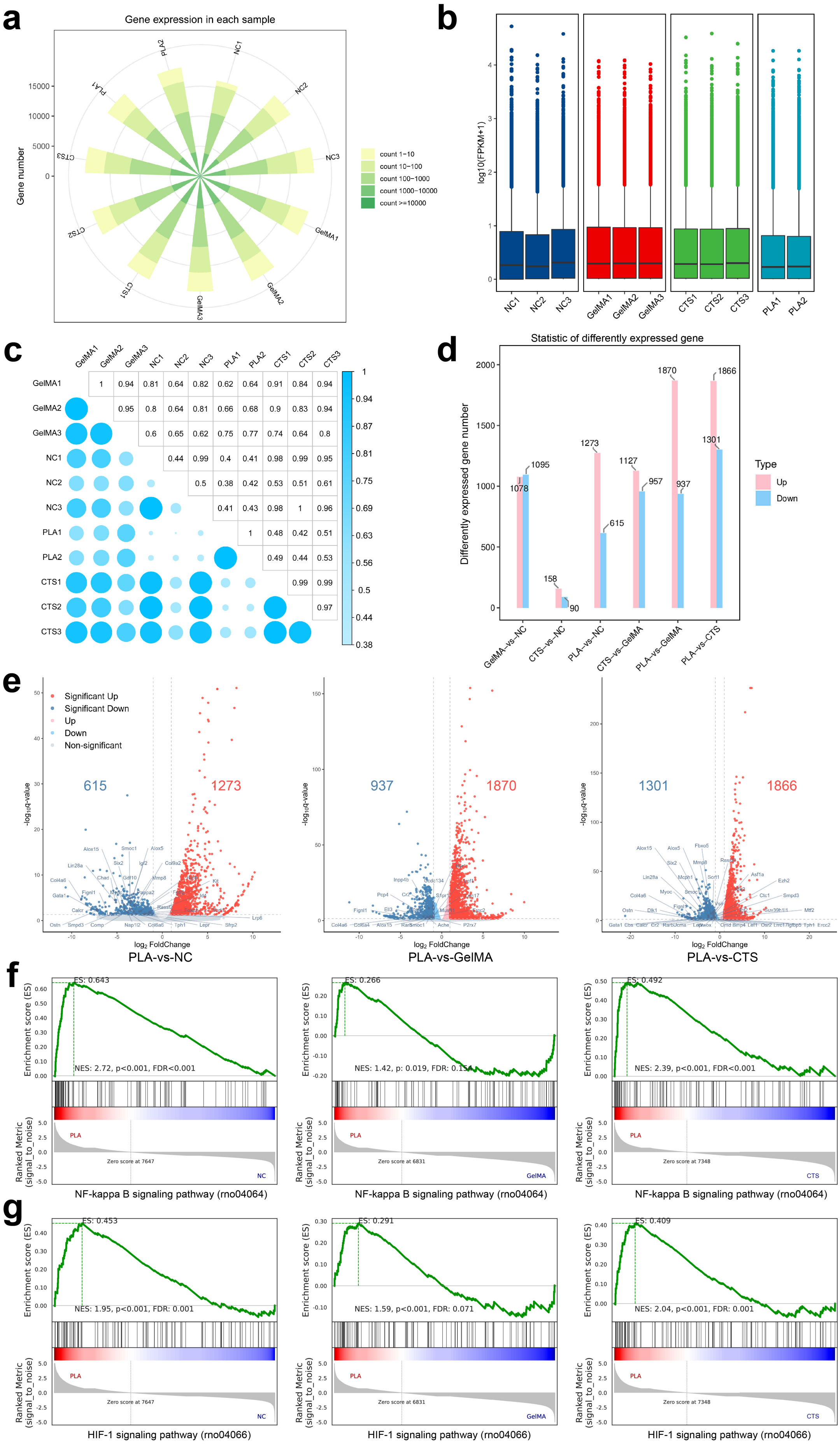

a
Gene expression in each sample
Gene number
15000
10000
5000
0
PLA2
NC1
NC2
NC3
GelMA1
GelMA2
GelMA3
CTS1
CTS2
CTS3
PLA1
count 1~10
count 10~100
count 100~1000
count 1000~10000
count >=10000
b
log10(FPKM+1)
NC1
NC2
NC3
GelMA1
GelMA2
GelMA3
CTS1
CTS2
CTS3
PLA1
PLA2
c
GelMA1 GelMA2 GelMA3 NC1 NC2 NC3 PLA1 PLA2 CTS1 CTS2 CTS3
GelMA1 1 0.94 0.81 0.64 0.82 0.62 0.64 0.91 0.84 0.94
GelMA2 0.95 0.8 0.64 0.81 0.66 0.68 0.9 0.83 0.94
GelMA3 0.6 0.65 0.62 0.75 0.77 0.74 0.64 0.8
NC1 0.44 0.99 0.4 0.41 0.98 0.99 0.95
NC2 0.5 0.38 0.42 0.53 0.51 0.61
NC3 0.41 0.43 0.98 1 0.96
PLA1 1 0.48 0.42 0.51
PLA2 0.49 0.44 0.53
CTS1 0.99 0.99
CTS2 0.97
CTS3
1
0.94
0.87
0.81
0.75
0.69
0.63
0.56
0.5
0.44
0.38
d
Statistic of differently expressed gene
Differently expressed gene number
1078
1095
158
90
1273
615
1127
957
1870
937
1866
1301
GelMA−vs−NC
CTS−vs−NC
PLA−vs−NC
CTS−vs−GelMA
PLA−vs−GelMA
PLA−vs−CTS
Type
Up
Down
e
Significant Up
Significant Down
Up
Down
Non-significant
615
1273
937
1870
1301
1866
-log10q-value
log2 FoldChange
PLA-vs-NC
PLA-vs-GelMA
PLA-vs-CTS
f
Enrichment score (ES)
ES: 0.643
NES: 2.72, p<0.001, FDR<0.001
ES: 0.266
NES: 1.42, p: 0.019, FDR: 0.154
ES: 0.492
NES: 2.39, p<0.001, FDR<0.001
Ranked Metric (signal_to_noise)
PLA
Zero score at 7647
NC
Zero score at 6831
GelMA
Zero score at 7348
CTS
NF-kappa B signaling pathway (rno04064)
g
ES: 0.453
NES: 1.95, p<0.001, FDR: 0.001
ES: 0.291
NES: 1.59, p<0.001, FDR: 0.071
ES: 0.409
NES: 2.04, p<0.001, FDR: 0.001
HIF-1 signaling pathway (rno04066)

Supplementary Fig. 5 Supplemental information on RNA-seq. **a** Radial bar chart displaying gene expression in each sample. **b** Boxplot of FPKM distribution among samples. **c** Sample correlation heatmap. **d** Bar chart showing numbers of upregulated and downregulated differentially expressed genes (DEGs) in the six comparison groups (GelMA-vs-NC, CTS-vs-NC, PLA-vs-NC, CTS-vs-GelMA, PLA-vs-GelMA, and PLA-vs-CTS). **e** Volcano plot illustrating the DEGs in PLA compared with NC, GelMA, and CTS. The downregulated DEGs associated with ossification are labeled. (**e**) is related to Fig. 4a, b. **f, g** GESA analysis demonstrating activated NF-κB (**f**) and HIF-1 (**g**) signaling pathways by PLA.

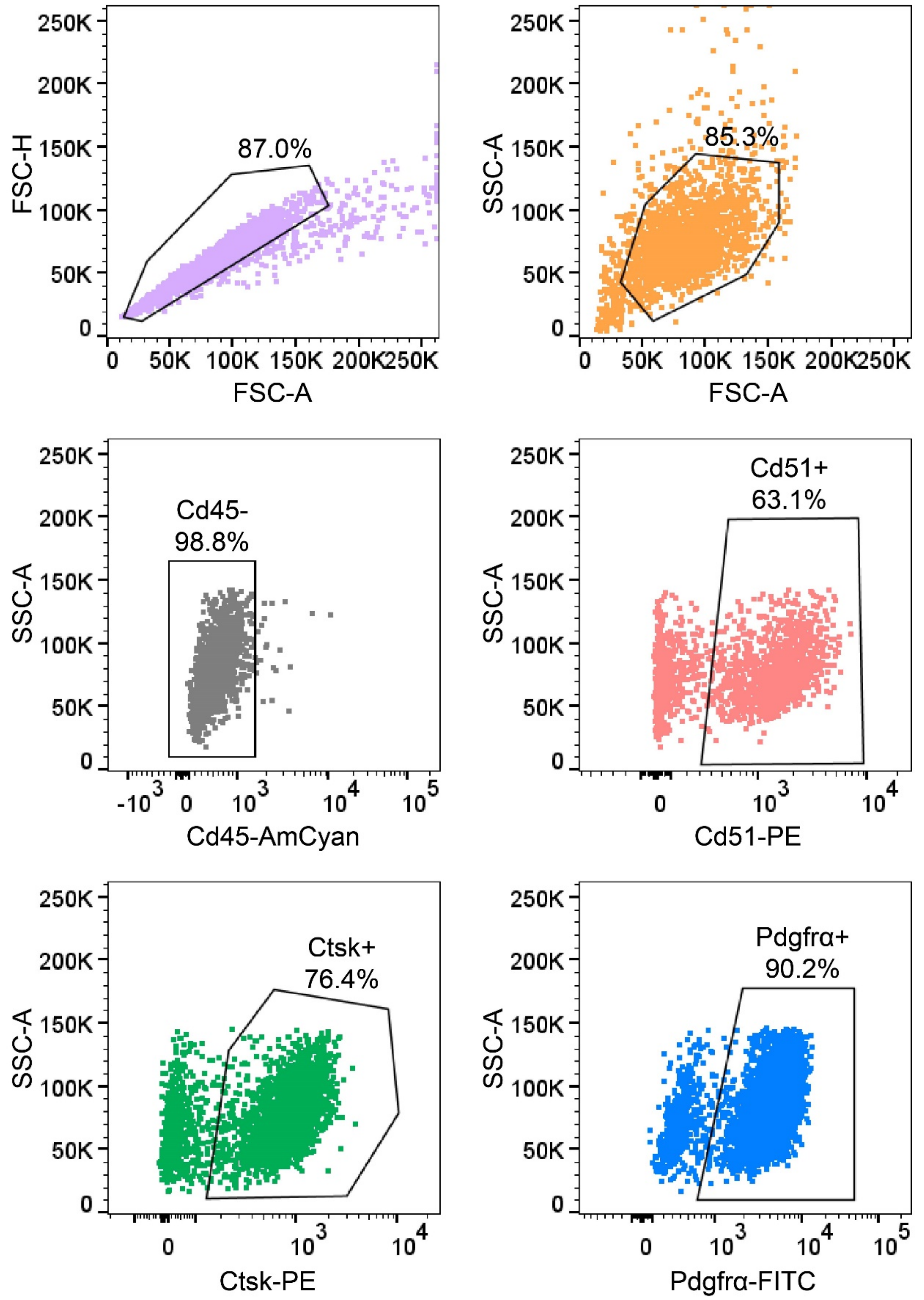

Supplementary Fig. 6 Gating strategy for FCM analysis related to Figure 7i.

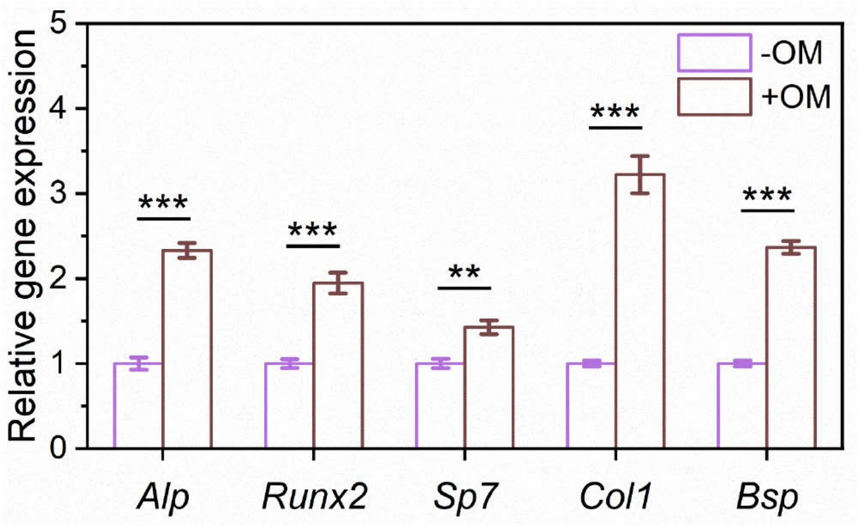

Supplementary Fig. 7 MSC were cultured in osteogenic medium (OM) for 5 days to obtain OB. RT-qPCR detecting osteogenic gene expressions in MSC with (+OM) or without (-OM) osteogenic induction. Each sample was examined in triplicate. *Gapdh* was used as the internal control. Data are expressed as mean ± SD. ***p<0.01; ****p<0.001.

Supplemental Tables

Supplementary Table 1 Specific primers of osteogenic-related genes in Rats

**Gene name**		**Sequence**
Gapdh	Forward	GCATCTTCTTGTGCAGTGCC
	Reverse	GATGGTGATGGGTTTCCCGT
Alp	Forward	TGCCTACTTGTGTGGCGTGAA
	Reverse	TCACCCGAGTGGTAGTCACAATG
Runx2	Forward	ATGCTTCATTCGCCTCACAAA
	Reverse	GCACTCACTGACTCGGTTGG
Sp7	Forward	GCCAGTAATCTTCGTGCCAG
	Reverse	GGACTGGAGCCATAGTGAGC
Col1	Forward	GGATCGACCCTAACCAAGGC
	Reverse	GATCGGAACCTTCGCTTCCA
Bsp	Forward	CAGGACTGCCGAAGGAAGAAA
	Reverse	TCGAGAAAGCACTCGCCATC
Mepe	Forward	TGTCTGTTGGACTGTTCCT
	Reverse	CCATCTTCATTCAGCTTTGG
Phex	Forward	AAACCCGCAAGTATTTAGCA
	Reverse	TTGGGTTTGTAAACCACTCTG

Supplementary Table 2 Antibodies in this study

Antibody	brand	Cat no.	Use	Condition
CD200 (E5I9V) XP® Rabbit mAb	Cell Signaling	Cat#23451	IF	1:100
Integrin αV/ITGAV/CD51 Antibody (H-2)	Santa Cruz	Cat#sc-376156	IF	1:40
Human Osterix/Sp7 MAb (Clone 764704)	R&D	Cat#MAB7547	IF	1:75
Rabbit polyclonal to Ki67	Abcam	Cat#ab15580	IF	1:200
Rabbit polyclonal anti-Cathepsin K	Abcam	Cat#ab19027	IF<br>FCM	1:100<br>1:75
CD45 (clone 30-F11) Brilliant Violet 510™	BioLegend	Cat#103138	FCM	1:50
Rabbit monoclonal to Integrin alpha V	Abcam	ab179475	FCM	1:100
PDGF Receptor alpha/PDGFRA Antibody (C-9)	Santa Cruz	Cat#sc-398206	FCM	1:20
Alexa Fluor® 488 Anti-S100A4 antibody [EPR2761(2)]	Abcam	Cat#ab196380	IF	1:150
Goat anti-Rabbit IgG-H&L (Alexa Fluor® 488)	Abcam	Cat#ab150077	IF	1:200
Alexa Fluor 555-labeled Donkey Anti-Rabbit IgG(H+L)	Beyotime	Cat#A0453	IF	1:200
Goat anti-mouse IgG-H&L (Alexa Fluor® 488)	Abcam	Cat#ab150113	IF<br>FCM	1:200<br>1:2000
Goat anti-Rabbit IgG-H&L (Alexa Fluor® 594)	Abcam	Cat#ab150080	FCM	1:2000

Supplementary Table 3 Key resources table

Reagent or Resource	Source	Identifier
Gelatin from porcine skin	Sigma-Aldrich	Cat#V900863
Methacrylic anhydride	Aladdin	Cat#M102519
Lithium phenyl-2,4,6-trimethylbenzoylphosphinate (LAP)	Sigma-Aldrich	Cat#900889
Chitosan	Aladdin	Cat#C105802
Sodium tripolyphosphate	Aladdin	Cat#S100099
Acetic acid	Aladdin	Cat#A298827
N, N-Dimethylformamide	Aladdin	Cat#D111999
Dichloromethane	Aladdin	Cat#D433567
4% paraformaldehyde	Biosharp	Cat#BL539A
Hematoxylin and Eosin (H&E) staining kit	Biosharp	Cat#BL700B
Masson's trichrome staining kit	Solarbio	Cat#G1340
Triton X-100	Thermo Scientific	Cat#85111
Albumin Bovine (BSA)	BioFroxx	Cat#4240GR100
Alpha-modified Eagle's medium (αMEM)	HyClone	Cat#SH30265
Fetal bovine serum (FBS)	Gibco	Cat#10091155
Penicillin-streptomycin	Gibco	Cat#15140122
β-glycerophosphate	Sigma-Aldrich	Cat#G9422
L-ascorbic acid	Sigma-Aldrich	Cat#A4403
Dexamethasone	Sigma-Aldrich	Cat#D4902
Cell counting kit-8 (CCK-8)	KeyGEN BioTECH	Cat#KGA317
Annexin V-PE/7-AAD Apoptosis Detection Kit	KeyGEN BioTECH	Cat#KGA1015-KGA1018
HiScript III RT SuperMix for qPCR (+gDNA wiper)	Vazyme	Cat#R323-01
ChamQ Universal SYBR qPCR Master Mix	Vazyme	Cat#Q511-0

TRIzolTM	Invitrogen	Cat#15596026
Collagenase, Type 1	BioFroxx	Cat#1904MG100
Ethylene Diamine Tetraacetic Acid (EDTA)	BioFroxx	Cat#1340GR500
Red Cell Lysis Buffer	Solarbio	Cat#R1010
BD Cytofix/Cytoperm™	Becton Dickinson (BD)	Cat#554722
